# Multidrug-resistant Commensal *Escherichia coli* in Children, Peru and Bolivia

**DOI:** 10.3201/eid1206.051258

**Published:** 2006-06

**Authors:** Alessandro Bartoloni, Lucia Pallecchi, Marta Benedetti, Connie Fernandez, Yolanda Vallejos, Elisa Guzman, Ana Liz Villagran, Antonia Mantella, Chiara Lucchetti, Filippo Bartalesi, Marianne Strohmeyer, Angela Bechini, Herlan Gamboa, Hugo Rodríguez, Torkel Falkenberg, Göran Kronvall, Eduardo Gotuzzo, Franco Paradisi, Gian Maria Rossolini

**Affiliations:** * Università di Firenze, Florence, Italy;; † Università di Siena, Siena, Italy;; ‡ Hospital Apoyo Yurimaguas, Yurimaguas–Loreto, Peru;; § Servicio Departamental de Salud Santa Cruz, Camiri, Bolivia;; ¶ Hospital Moyobamba, Moyobamba–San Martin, Peru;; # Karolinska Institute, Stockholm, Sweden;; ** Universidad Peruana Cayetano Heredia, Lima, Peru

**Keywords:** Escherichia coli, antimicrobial resistance, commensal bacteria, resistance surveillance, Peru, Bolivia, research

## Abstract

Using a rapid screening method, we investigated the prevalence of fecal carriage of antimicrobial drug–resistant *Escherichia coli* in 3,174 healthy children from 4 urban settings in Peru and Bolivia. High resistance rates were observed for ampicillin (95%), trimethoprim-sulfamethoxazole (94%), tetracycline (93%), streptomycin (82%), and chloramphenicol (70%). Lower resistance rates were observed for nalidixic acid (35%), kanamycin (28%), gentamicin (21%), and ciprofloxacin (18%); resistance to ceftriaxone and amikacin was uncommon (<0.5%). In a random sample of 1,080 resistant *E. coli* isolates, 90% exhibited a multidrug-resistance (MDR) phenotype. The 2 most common MDR phenotypes (ampicillin/tetracycline/trimethoprim-sulfamethoxazole and ampicillin/tetracycline/trimethoprim-sulfamethoxazole/chloramphenicol) could be transferred en bloc in conjugation experiments. The most common acquired resistance genes were *bla*_TEM_, *tet*(A), *tet*(B), *drfA8*, *sul1*, *sul2*, and *catI*. These findings underscore the magnitude of the problem of antimicrobial drug resistance in low-resource settings and the urgent need for surveillance and control of this phenomenon.

## Abstract 2

Healthy children in urban areas have a high prevalence of fecal carriage of drug-resistant *E. coli.*

The spread of microbial drug resistance is a global public health challenge, which impairs the efficacy of antimicrobial agents and results in substantial increased illness and death rates and healthcare-associated costs (*1**–**3*). In low-resource countries, the extent and the impact of the phenomenon tend to be even larger than in industrialized countries. In fact, high resistance rates have often been reported in surveillance studies dealing with clinical isolates (*1**,**4**,**5*) and in prevalence studies of commensal bacteria taken as indicators to estimate spread of acquired resistance (*6**–**15*). Moreover, in low-resource countries the impact of antimicrobial drug resistance on illness and death rates tends to be greater because of the high prevalence of bacterial infections and the major role of antimicrobial agents in combating infectious diseases (*1**,**3**,**4**,**16**,**17*). The high antimicrobial drug resistance rates observed in low-resource countries are likely due to a combination of several factors, among which irrational antimicrobial drug usage and conditions of poor sanitation are thought to play a major role, even if the relative importance of additional factors remains unclear (*1**,**4**,**8**,**9*).

ANTRES (Towards Controlling Antimicrobial Use and Resistance in Low-Income Countries: An intervention Study in Latin America) is a research project that aims to investigate this phenomenon on a large scale. The project is carried out in 2 Latin American countries, Bolivia and Peru, where 4 urban areas have been selected for studying antimicrobial drug use and bacterial resistance in healthy children by a prospective approach. An information-education-communication strategy will be developed based on the collected information and involving local health services. The impact on antimicrobial drug use and bacterial resistance trends will be evaluated (http://www.unifi.it/infdis/antres/default.htm).

We report the results of the baseline study, carried out at the beginning of the project, to assess the antimicrobial drug resistance rates in the studied areas. Very high resistance rates to several antimicrobial agents were observed in commensal bacteria from the population of each area. Strains showing multidrug-resistance (MDR) phenotypes were widely disseminated.

## Materials and Methods

### Study Design and Population

The study population was represented by healthy children 6–72 months of age from 4 urban areas, 2 in Bolivia (Camiri, Santa Cruz Department; Villa Montes, Tarija Department) and 2 in Peru (Yurimaguas, Loreto Department; Moyobamba, San Martin Department). The urban areas were communities of ≈25,000 to 30,000 inhabitants who had comparable demographic and socioeconomic characteristics. Eight hundred children from each area were enrolled in the study to cover at least 25% of all households with children in the target age cohort. Only children who had not had diarrhea (as defined by the World Health Organization [*18*]) during the previous 24 hours were eligible for inclusion in the study. In each household, the youngest recruitable child in the target age cohort was selected. The studied households were selected with a modified cluster sampling: detailed maps outlining the distribution of households were obtained from each study area, and each city was divided into 80 small clusters. In each cluster a number of households selected at random were visited until 10 children were reached. A rectal swab was obtained from each child enrolled in the study, after informed consent was obtained from parents or other legal guardians. Before the sample was obtained, adult members of the household were interviewed to collect information on the family's socioeconomic and cultural setting, the household antimicrobial drug use, and the health status of the selected child. In each country, full ethical clearance was obtained from the qualified authorities who had revised and approved the study design.

**Figure 1:**
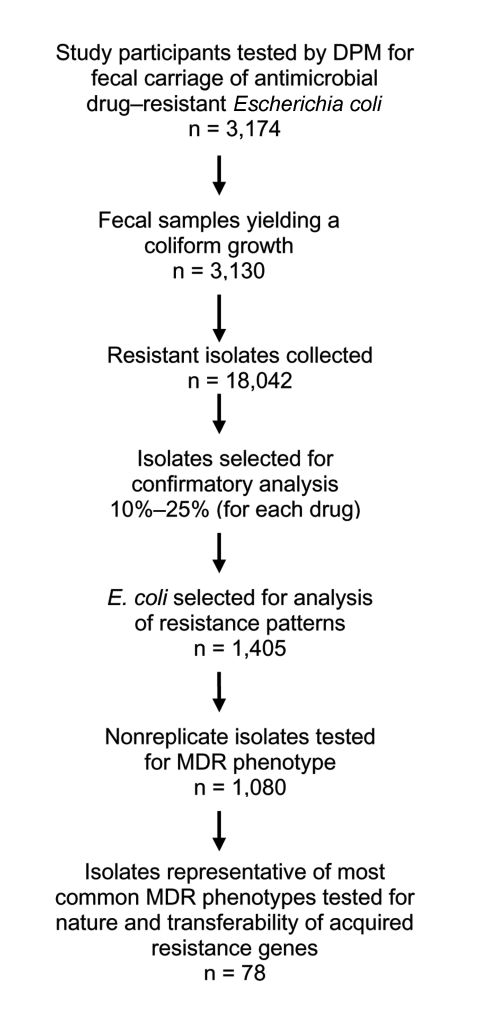
Flow chart of microbiologic analysis of fecal samples. DPM, direct plating method.

The overall acceptance rate was >95% in all studied areas. Rectal swabs were obtained from a total of 3,174 children (Figure 1): 794 from Camiri and 790 from Villa Montes, Bolivia; 797 from Yurimaguas, and 793 from Moyobamba, Peru. The study participants were 6–72 months of age (mean 34.7 ± 18.2 months; median age 33.5 months).The female:male ratio was 0.95. No significant differences in age or sex were observed among children enrolled from the different areas. The study was carried out for 4 months (August–November 2002). In each area, the sampling period was not longer than 7 weeks.

### Screening for Resistant *Escherichia coli* in Commensal Microbiota

Rectal swabs, stored in Amies transport medium (Oxoid, Milan, Italy), were transferred in a cold box to the laboratory of the corresponding district hospital within 3 hours of collection. The 4 laboratories participate in national quality control programs. A training phase and a pilot study preceded the survey. Three European investigators (A.B., M.B., L.P.) participated in the sample analysis in the 4 laboratories. This approach was chosen to limit variability in the microbiologic procedures during the field studies.

Screening for antimicrobial drug-resistant *E. coli* in the fecal microbiota was carried out by a direct plating method, as described previously (*7**,**19*). This method is preferred because it correlates well with methods based on testing of randomly collected colonies from primary stool culture, but it is more sensitive (*11**,**19*).

Briefly, each swab was spread on a McConkey agar no. 3 plate (Oxoid) to yield uniform growth, and antimicrobial drug–containing disks were directly placed onto the seeded plate. Antimicrobial agents tested included ampicillin, ceftriaxone, tetracycline, trimethoprim-sulfamethoxazole, chloramphenicol, streptomycin, kanamycin, gentamicin, amikacin, nalidixic acid, and ciprofloxacin (Oxoid). After incubation at 37°C for 12 to 14 hours, plates were inspected for growth, and inhibition zone diameters were measured. The presence of a growth inhibition zone larger than the established breakpoint diameter was considered to indicate susceptibility to that agent. The presence of a growth inhibition zone smaller than the breakpoint diameter, the absence of any inhibition zone, or the presence of isolated colonies growing inside an inhibition zone of any size was considered indicative of resistance. In the latter case, however, resistance was considered not to be represented in the dominant flora. Breakpoints were as previously described for ampicillin, tetracycline, trimethoprim-sulfamethoxazole, chloramphenicol, amikacin, nalidixic acid, and ciprofloxacin (*19*), and 14 mm, 12 mm, 12 mm, and 14 mm for kanamycin, streptomycin, gentamicin, and ceftriaxone, respectively (A. Bartoloni, unpub. data). Only bacterial growth exhibiting a shape typical of *E. coli* was considered valid for the analysis. Specially prepared forms were used to record microbiologic results, including growth features and susceptibility patterns. Confluent bacterial growth was obtained from 3,130 (99%) of the 3,174 collected rectal swabs (Figure 1). In a few cases insufficient growth (11 swabs) or noncoliform growth (33 swabs) was observed. From each plate and for each drug, the putatively resistant coliform growth (i.e., a pool of the colonies grown inside the zone of inhibition or a loopful of the microbial lawn grown in proximity of the disk) were collected (n = 18,042) (Figure 1). Of these, a random sample (10%–25% for each drug, depending on the prevalence of the resistance phenotype) were subjected to confirmatory analysis for resistant *E. coli* (Figure 1) by using the API20E identification system (bioMérieux, Marcy l'Étoile, France) and the standard agar disk diffusion method (*20**,**21*). Confirmation was obtained in >96% of the tested samples, without significant differences among the 4 laboratories.

### Analysis of Resistance Patterns of Commensal *E. coli*

Susceptibility to several antimicrobial agents representative of various classes (ampicillin, tetracycline, trimethoprim-sulfamethoxazole, chloramphenicol, kanamycin, gentamicin, nalidixic acid, ciprofloxacin) was tested in 1,405 (8%) *E. coli* isolates randomly selected from the 18,042 collected samples. Each isolate was spread onto Mueller-Hinton (MH) agar plates containing each antimicrobial agent at a concentration 20% higher than the breakpoints for resistance of each antimicrobial agent, as per National Committee of Laboratory Standards guidelines (*20**,**21*). The inoculum size was ≈2 × 10^5^ CFU per spot. A plate of antimicrobial drug–free medium was always included as a growth control. *E. coli* ATCC 25922, and *E. coli* strains from our collection resistant to the various antimicrobial agents used in the assay were always included for quality control purposes. Results were recorded after incubation at 37°C for 18 hours. A resistance phenotype was assigned when growth was observed on the medium containing an antimicrobial agent. An MDR phenotype was intended as resistance to >2 classes of antimicrobial agents. Isolates from the same study participant that exhibited the same resistance phenotype (the random sampling procedure did not initially consider the study participant source) were considered replicated isolates and were counted only once for data analysis. According to this criterion, data analysis was conducted with 1,080 isolates (Figure 1).

Data entry and analysis were calculated with the EpiInfo software package (version 2002, Centers for Disease Control and Prevention, Atlanta, GA, USA). Statistical differences in the prevalence of antimicrobial drug resistances were determined by the χ^2^ test.

### Molecular Analysis of Resistance Genes and Conjugation Assay

Acquired resistance genes and conjugative transfer of resistance traits were investigated in 78 isolates randomly selected from those representative of the 2 most common MDR phenotypes (Figure 1). Resistance genes *bla*_TEM_, *catI*, *dfrA8*, *sul1*, and *sul2* and the *intI1* integrase gene were detected by colony blot hybridization (*22*) with specific probes generated by polymerase chain reaction (PCR), as described previously (*23**–**25*). Tetracycline resistance genes *tet*(A), *tet*(B), *tet*(C), and *tet*(D) were detected by PCR, as described previously (*26*). Conjugative transfer of resistance genes was assayed in MH broth using *E. coli* J53 (auxotropic for proline and methionine and resistant to rifampin and nalidixic acid) as a recipient and an initial donor/recipient ratio of 0.1. Mating tubes were incubated at 30°C for 14 h. Transconjugants were selected on MH agar containing rifampin (400 μg/mL) and nalidixic acid (32 μg/mL) plus 1 of the following antimicrobial agents: ampicillin (200 μg/mL), tetracycline (5 μg/mL), chloramphenicol (30 μg/mL), or trimethoprim-sulfamethoxazole (40/200 μg/mL). Under the above conditions, the detection sensitivity of the mating assay was >1 × 10^8^ transconjugants/recipients.

## Results

### Antimicrobial Drug–Resistance Rates

Overall, high resistance rates were observed for ampicillin (95%), trimethoprim-sulfamethoxazole (94%), tetracycline (93%), streptomycin (82%), and chloramphenicol (70%). Lower resistance rates were observed for nalidixic acid (35%), kanamycin (28%), gentamicin (21%), and ciprofloxacin (18%). Resistance to ceftriaxone and amikacin was uncommon (<0.5%) (Table 1). For first-line oral antimicrobial agents such as ampicillin, tetracycline, and trimethoprim-sulfamethoxazole, resistant strains were present as the dominant flora in >80% of carriers (Table 1).

**Table 1:** Prevalence, expressed as percentage, of healthy children carrying antimicrobial drug–resistant *Escherichia coli* as part of their commensal flora and of children in whom resistant *E. coli* constituted the predominant flora*†

Drug	Bolivia			Peru						
	Camiri	Villa Montes	p value	Yurimaguas	Moyobamba	p value	Bolivia, subtotal	Peru, subtotal	Total	p value
Ampicillin	98 (95)	96 (87)	<0.05	92 (76)	93 (83)	NS	97 (91)	92 (80)	95 (85)	<0.001
SXT‡	98 (94)	95 (86)	<0.05	89 (72)	93 (83)	<0.05	96 (90)	91 (77)	94 (84)	<0.001
Tetracycline	96 (91)	92 (82)	<0.05	89 (71)	93 (81)	<0.05	94 (86)	91 (76)	93 (81)	<0.05
Streptomycin	96 (89)	88 (79)	<0.001	71 (50)	86 (66)	<0.001	92 (84)	79 (58)	82 (68)	<0.001
Chloramphenicol	74 (58)	65 (40)	<0.001	69 (42)	72 (48)	NS	70 (49)	71 (45)	70 (47)	NS
Nalidixic acid	44 (25)	29 (13)	<0.001	27 (8)	41 (18)	<0.001	36 (19)	38 (13)	35 (16)	NS
Kanamycin	37 (20)	31 (12)	<0.05	22 (8)	23 (9)	NS	34 (16)	22 (9)	28 (12)	<0.001
Gentamicin	28 (16)	18 (10)	<0.001	19 (9)	21 (10)	NS	23 (13)	20 (10)	21 (11)	<0.05
Ciprofloxacin	21 (10)	10 (4)	<0.001	16 (5)	25 (9)	<0.001	16 (7)	21 (7)	18 (7)	<0.001
Amikacin	0	0.1 (0)	NA	1 (0.4)	0.4 (0.3)	NA	0.1 (0.1)	1 (0.3)	0.4 (0.2)	NA
Ceftriaxone	0.1 (0)	0.1 (0.1)	NA	0.3 (0)	0.1 (0.1)	NA	0.1 (0.1)	0.1 (0.1)	0.1 (0.1)	NA

*Inside parentheses: proportion of carriers in whom resistant *E. coli* constituted the predominant flora. NS, not significant; NA, not applicable. †Number of children from whom samples were obtained: Camiri = 794; Villa Montes = 790; Yurimaguas = 797; Moyobamba = 793; Bolivia = 1,584; Peru = 1,590; and total = 3,174. ‡SXT, trimethoprim-sulfamethoxazole.

Resistance rates for ampicillin, trimethoprim-sulfamethoxazole, kanamycin, and streptomycin were significantly higher in Bolivia than in Peru (p<0.001), whereas ciprofloxacin resistance rates were significantly higher in Peru than in Bolivia (p<0.001) (Table 1). Differences in the resistance rates were also observed within each country. In Bolivia, higher overall resistance rates were found in Camiri than in Villa Montes (p<0.001) for chloramphenicol, streptomycin, gentamicin, nalidixic acid, and ciprofloxacin (Table 1). In Peru, higher overall resistance rates were found in Moyobamba than in Yurimaguas. The most significant differences (p<0.001) were noted for streptomycin, nalidixic acid, and ciprofloxacin (Table 1).

**Figure 2:**
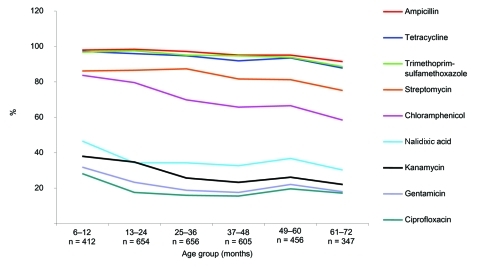
Total prevalence, by age group, of fecal carriage of antimicrobial drug–resistant *Escherichia coli* among 3,174 children in 4 urban areas of Bolivia and Peru. Ceftriaxone and amikacin were not considered in these analyses because their resistance rates were too low.

Significantly higher (p<0.05) resistant rates were observed in boys, with some agents, and in some settings (ampicillin in both Peruvian cities, trimethoprim-sulfamethoxazole in Moyobamba, and chloramphenicol, kanamycin, and gentamicin in Camiri). Analysis by age showed that, with all agents (ceftriaxone and amikacin were not considered in this analysis because of the low numbers of resistant isolates), resistance rates were notably higher in the youngest age group, and an overall decreasing trend by age was observed (Figure 2). With some agents (kanamycin, gentamicin, and the quinolones), the decreasing trend was essentially limited to the younger age groups; with other agents (ampicillin, tetracycline, trimethoprim-sulfamethoxazole, and chloramphenicol); this phenomenon was more evident in the older age groups.

### Combinations of Antimicrobial Drug–resistance Traits in Healthy Children

Of the 3,174 children evaluated in the study, only 84 (2.7%) carried an *E. coli* fecal population susceptible to all the antimicrobial agents tested, while 46 (1.5%), 73 (2.3%), 187 (5.9%), 537 (17%), 808 (26%), 624 (20%), and 816 (26%) had an *E. coli* population resistant to 1, 2, 3, 4, 5, 6, or >6 antimicrobial agents. No significant differences in this distribution were observed between the 2 countries. Of the 156 different combinations of resistance observed, 2 were most prevalent: 1) the pattern with 4 resistances (ampicillin, tetracycline, trimethoprim-sulfamethoxazole, and streptomycin) in 361 (11.5%) children; and 2) the pattern with 5 resistances (ampicillin, tetracycline, trimethoprim-sulfamethoxazole, streptomycin, and chloramphenicol) in 567 (18%) children.

### Patterns of Resistance Phenotypes of *E. coli* Isolates

**Figure 3:**
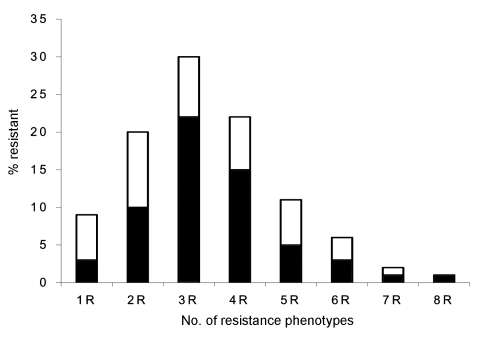
Frequency of resistance phenotypes in 1,080 randomly selected antimicrobial drug–resistant *Escherichia coli* isolates from 4 urban areas of Bolivia and Peru. Black bars indicate the most frequent resistance and multidrug-resistance phenotype within each category: 1R, TET; 2R, AMP-SXT; 3R, AMP-TET-SXT; 4R, AMP-TET-SXT-CHL; 5R, AMP-TET-SXT-CHL-KAN; 6R, AMP-TET-SXT-CHL-NAL-CIP; 7R, AMP-TET-SXT-CHL-GEN-NAL-CIP; 8R, AMP-TET-SXT-CHL-KAN-GEN-NAL-CIP. AMP, ampicillin; TET, tetracycline; SXT, trimethoprim-sulfamethoxazole; CHL, chloramphenicol; KAN, kanamycin; GEN, gentamicin; NAL, nalidixic acid; CIP, ciprofloxacin.

Frequency and patterns of resistance phenotypes were determined on a random sample of 1,080 nonreplicate resistant *E. coli* isolates with 8 antimicrobial agents representative of 6 different classes (ampicillin for β-lactams, tetracycline for tetracyclines, chloramphenicol for phenicols, trimethoprim-sulfamethoxazole for folate inhibitors, kanamycin and gentamicin for aminoglycosides, and nalidixic acid and ciprofloxacin for quinolones). Only a few isolates were resistant to a single drug (9%) (Figure 3). Isolates showing resistance to 3 different drugs were the most prevalent (30%), followed by those showing resistance to 4 (22%) and 2 (20%) drugs, respectively. Seven isolates (<1%) showed resistance to all tested drugs (Figure 3). An MDR phenotype was common (90% of isolates). Resistance patterns were quite variable. Of 97 different patterns observed, some were highly prevalent. The pattern that included ampicillin, tetracycline, and trimethoprim-sulfamethoxazole was the most prevalent, followed by that including ampicillin, tetracycline, trimethoprim-sulfamethoxazole, and chloramphenicol, as well as that including ampicillin and trimethoprim-sulfamethoxazole alone (22%, 15%, and 10% of the tested isolates, respectively) (Figure 3).

### Acquired Resistance Genes and Transferability of Resistance Traits

The prevalence of several acquired resistance genes (*bla*_TEM_ for ampicillin resistance; *tet*(A)–(D) for tetracycline resistance; *dfrA8* for trimethoprim resistance; *sul1* and *sul2* for sulfonamide resistance; *catI* for chloramphenicol resistance) and class 1 integrons was investigated in 78 isolates randomly selected from representatives of the 2 most prevalent MDR phenotypes (ampicillin/tetracycline/trimethoprim-sulfamethoxazole, n = 45; ampicillin/tetracycline/trimethoprim-sulfamethoxazole/chloramphenicol, n = 33). In most cases the resistance phenotype could be accounted for by a combination of the above resistance genes, and some of them appeared to be highly prevalent (Table 2). The presence of the *intI1* integrase gene (associated with class 1 integrons) was detected in 35% of MDR isolates.

**Table 2:** Acquired resistance genes in 78 MDR commensal Escherichia coli*

Resistance trait	No. resistant isolates	Resistance gene	No. (%)† positive isolates
Ampicillin	78	*bla*_TEM_	77 (99)
Tetracycline	78	*tet*(A)	27 (35)
		*tet*(B)	44 (56)
		*tet*(D)	4 (5)
		*tet*(A) and *tet*(B)	1 (1)
Trimethoprim	78	*dfrA8*	42 (54)
Sulfamethoxazole	78	*sul1*	7 (9)
		*sul2*	54 (69)
		*sul1* and *sul2*	17 (22)
Chloramphenicol	33	*catI*	33 (100)

*MDR, multidrug resistant. †Percentages refer to positive isolates among isolates resistant to each antimicrobial agent.

These 78 isolates were also tested for transferability of resistance traits in conjugation experiments. Overall, the transfer of at least 1 resistant trait was observed in 37 cases (47%). Transfer rates for each resistance trait were as follows: ampicillin 46%, tetracycline 40%, trimethoprim-sulfamethoxazole 41%, and chloramphenicol 27%. Co-transfer of all the resistance traits was observed in 26 of the 37 cases that gave positive results in the conjugation experiments. Molecular analysis of the transconjugants showed that, in all the cases, the resistance phenotype could be accounted for by the acquisition of resistance genes detected in the respective donors. Conjugative transfer of class 1 integrons was observed in 10 (37%) of the 27 *intI1–*positive isolates.

## Discussion

Similar to pathogenic bacteria, commensals are exposed to the selective pressure of antimicrobial agents, and commensal *E. coli* have often been used as an indicator of the dissemination of acquired resistance genes (*7**,**8**,**11**–**13**,**15**,**27*). To our knowledge, this study is the first to address the status of antimicrobial drug resistance in commensal *E. coli* in a large population of preschool-age children from urban settings of low-resource countries. Results showed a high prevalence (70%–95%) of healthy carriers of *E. coli* resistant to a number of older antimicrobial agents (ampicillin, tetracycline, trimethoprim-sulfamethoxazole, chloramphenicol, and streptomycin). For these drugs, the small differences observed among localities, although sometimes statistically significant, are probably of limited clinical and epidemiologic relevance and presumably attributable to the large number of antimicrobial drug–resistant *E. coli* carriers. A relatively high prevalence (18%–35%) of carriers of *E. coli* resistant to other agents (kanamycin, gentamicin, nalidixic acid, and ciprofloxacin) was also observed, while the carriage of *E. coli* resistant to expanded-spectrum cephalosporins and amikacin was uncommon (<0.5%). Ranking patterns of resistance rates were similar overall in each of the 4 studied areas, which suggests a common scenario in urban areas of these 2 Latin American countries. This view is further supported by data recently published for adults from Lima (Peru), where similar resistance rates were reported (*12*).

Our findings were in substantial agreement with previous reports of high resistance rates in commensals of study participants from low-resource settings (*7**,**8**,**11**–**13**,**15**,**28*). Comparative analysis with previous data available for 1 studied setting (Camiri, Bolivia) surveyed 10 years earlier (*7*) indicated a significant increase in the resistance rates to gentamicin and nalidixic acid and the de novo appearance of resistance to ciprofloxacin.

One aspect that was not previously reported among preschool children was the age-related differences in resistance rates; rates were significantly higher in the younger age groups. This phenomenon could reflect a larger use of antimicrobial drugs in younger children. However, this explanation cannot be the case for quinolones, which are not prescribed in this age group. Another influencing factor could be dietary changes related to weaning. In fact, a diet high in milk that contains abundant lactoferrin, which chelates dietary iron, has been hypothesized to favor the presence of strains expressing iron-uptake systems (such as aerobactin or enterobactin), which are often encoded by plasmids that also carry resistance genes (*29*). However, further investigation will be necessary to clarify the mechanism responsible for this phenomenon.

Multiple resistance traits in the commensal *E. coli* microbiota were apparently the rule, as they were detected in most study participants. The simultaneous presence of multiple strains expressing different resistance phenotypes and single strains expressing MDR phenotypes could contribute to this phenomenon. Although the relative contribution of the 2 mechanisms was not specifically investigated, the high prevalence of strains expressing MDR phenotypes probably provides a major contribution. Some MDR patterns (e.g., ampicillin/tetracycline/trimethoprim-sulfamethoxazole, ampicillin/tetracycline/trimethoprim-sulfamethoxazole/chloramphenicol) were common and could be accounted for by a number of known acquired resistance genes. Resistance traits could be transferred by conjugation, often en bloc, suggesting a linkage of the corresponding resistance genes in self-transferable or mobilizable plasmids.

In conclusion, this study underscores the magnitude of the problem of antimicrobial drug resistance in low-resource settings and the urgent need for surveillance and control of this phenomenon. Inexpensive, sensitive, and simple methods to monitor antimicrobial drug resistance in commensal bacteria could be valuable tools for large-scale surveillance studies and to improve the efficacy of resistance control interventions. The direct plating method used in this study, which had previously shown high sensitivity and specificity in detecting resistant *E. coli* (*19*), was confirmed to be a valid tool to conduct such a large-scale survey.
